# Benzodiazepine and Zolpidem-Induced Bradycardia: A Case Report

**DOI:** 10.7759/cureus.52178

**Published:** 2024-01-12

**Authors:** Ivan Ivanov, Rachel Kirzhner, Brenda Sokup, Yan Sun

**Affiliations:** 1 Department of Emergency Medicine, NYC (New York City) Health + Hospitals/South Brooklyn Health, Brooklyn, USA; 2 Department of Resuscitation/Emergency Medicine, Stony Brook University, Stony Brook, USA

**Keywords:** zolpidem, bradycardia, clonazepam, alprazolam, toxicology, suicide, benzodiazepine

## Abstract

Benzodiazepines and zolpidem are commonly used in suicide attempts. They have previously been thought to have very few side effects outside of central nervous system depression, and are considered relatively safe even in overdoses. Cardiovascular manifestations of these overdoses are exceedingly rare. We depict a case where an unknown woman appearing to be in her 50s presented to our emergency department somnolent after a large ingestion of alprazolam, clonazepam, and zolpidem. During the evaluation, the patient became bradycardic to 35 beats per minute on the monitor, which responded to atropine administration. Therefore, it is important for an emergency medicine provider to be aware that both benzodiazepine and zolpidem overdoses can cause profound sinus bradycardia, which can respond to atropine.

## Introduction

Benzodiazepine abuse and overdose are becoming more common in the United States and across the world. Benzodiazepines were involved in 16.8% of overdose deaths between January 2019 and June 2020 in 23 states; however, opioids were also involved in a majority of these cases [1]. Oral overdoses of benzodiazepines can cause sedation and hypnosis; however, they rarely cause life-threatening hypoventilation [2]. Benzodiazepines have relative safety when ingested alone, even with substantial ingestions [2]. This is because benzodiazepines do not open gamma-aminobutyric acid (GABA) channels independently at high concentrations [2]. Benzodiazepines act by being a positive allosteric modulator, they bind between the alpha and gamma subunits of GABA-A receptors and cause a conformational change that allows GABA to bind and increases the frequency of opening of the chloride channel, which in turn hyperpolarizes the neuron and has an inhibitory effect [3]. Although CNS depression is commonly reported as a potential systemic toxicity to benzodiazepine overdoses, cardiovascular complications are exceedingly rare [4]. Zolpidem is an imidazopyridine that selectively binds the benzodiazepine receptor at ɑ1-GABAA receptors, and moderate activity at ɑ2-GABAA receptors and ɑ3-GABAA receptors, causing a sedative-hypnotic effect [5]. The most commonly reported adverse effects of zolpidem are nausea, dizziness, malaise, nightmares, agitation, headache, residual daytime sedation, confusion, disorientation, nervousness, amnesia, impaired concentration, and anxiety [6]. Some rarer adverse effects that have been noted are increased suicidality, complex behaviors in a sleep-like state like sleep-driving, hallucinations, psychosis, and even homicidality [7,8]. Although there have been some studies showing an increased risk of acute myocardial infarction with zolpidem use, cardiovascular complications are exceedingly rare [9]. We report a case where an intentional suicidal ingestion of alprazolam, clonazepam, and zolpidem induced bradycardia.

## Case presentation

**Figure 1:**
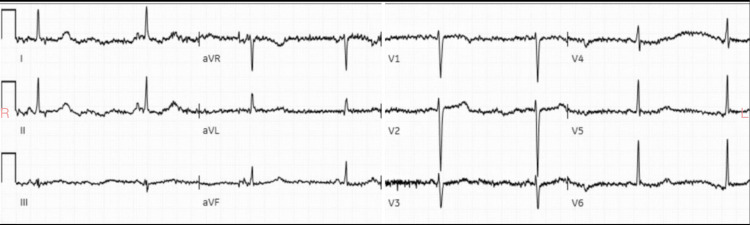
ECG on arrival showing sinus bradycardia

A woman who appeared to be in her 50s without any known medical history presented to our emergency department for altered mental status by emergency medical services (EMS). EMS reported that her family stated that she had a history of substance abuse, anxiety, and bipolar disorder. The family reported to EMS that she took one bottle of clonazepam, one bottle of alprazolam, and one bottle of zolpidem. How much was in each bottle was not precisely known. The patient had a prescription of 2 mg alprazolam filled within the last 30 days, while the dosage and pickup time of the clonazepam and zolpidem were both unknown. The exact time of ingestion was also not known; however, the patient had last been seen normal approximately 10 hours prior to presentation. The patient admitted to her family that she was trying to commit suicide. The family denied access to any other medications and were unsure if there was co-ingestion of alcohol or illicit substances. EMS noted the patient to be lethargic upon their arrival with stable vital signs and gave 2 milligrams of intranasal naloxone with minimal response. The patient presented to our emergency department somnolent, but arousable. She responded to physical stimulation and was able to voice a few words, although confused. The patient opened her eyes to loud verbal stimulation. The patient’s initial vital signs were a heart rate of 60 beats per minute, blood pressure of 100/64 mm Hg, respiratory rate of 16 breaths per minute, 100% saturation on room air, and a temperature of 97.4°F. End-tidal carbon dioxide was also placed and was noted to be 48 mm Hg with a normal waveform. The patient was brought to the resuscitation room and placed on the monitor and IV access was obtained. IV fluids were initiated. Point-of-care glucose was 93 mg/dL. The patient was protecting her airway and placement of an advanced airway device was not required. Pupils were equal, approximately 4 mm, and responsive to light without any nystagmus noted; and the patient was able to move all extremities. The patient was noted not to be diaphoretic or have any flushing of the skin. Mucous membranes were moist and no clonus, rigidity, or tremor was observed. As the physical exam progressed, the patient was noted to become more bradycardic. An initial ECG was obtained and is shown in Figure 1.

The patient was then noted to become even more bradycardic to a low of approximately 35 beats per minute with constant PR interval noted on the monitor. The patient’s mental status did not decline and her blood pressure remained approximately the same. The decision to give 1 mg of atropine intravenously was made due to the patient’s poor mental status and relative inability to explain if there was a minor decline. The patient’s heart rate responded and increased to approximately 70 beats per minute. The patient remained somnolent with a respiratory rate of 16 breaths per minute still. The decision was made not to give flumazenil due to possible chronic benzodiazepine use. Significant laboratory work-up is shown in Table 1.

**Table 1:** Significant laboratory values of the patient BMP: basic metabolic panel; VBG: venous blood gas; pCO2: partial pressure of carbon dioxide.

Lab	Value	Normal range
Troponin	<0.010 ng/mL	<0.010 ng/mL
Potassium	4.2 mmol/L	3.5-5.0 mmol/L
Bicarbonate (BMP)	28 mmol/L	22-29 mmol/L
pH	7.34	7.32-7.43
pCO2	66 mmHg	38-41 mmHg
Bicarbonate (VBG)	36 mmol/L	22-29 mmol/L
Thyroid-stimulating hormone	0.86 uIU/mL	0.27-4.20 uIU/mL
Acetaminophen level	<15 mcg/mL	0-30 mcg/mL
Salicylate level	<3 mg/dL	15-30 mg/dL
Digoxin level	<0.30 ng/dL	0.50-2.00 ng/dL
Ethanol level	<10 mg/dL	0-10 mg/dL
Urine barbiturate	Negative	<200 ng/mL
Urine benzodiazepines	Negative	<200 ng/mL
Urine cocaine	Negative	<300 ng/mL
Urine methadone	Negative	<300 ng/mL
Urine opiates	Negative	<300 ng/mL

The urine pregnancy test was negative and there was no evidence of infection on urinalysis. Chest X-ray was negative for acute pathology.

**Figure 2:**
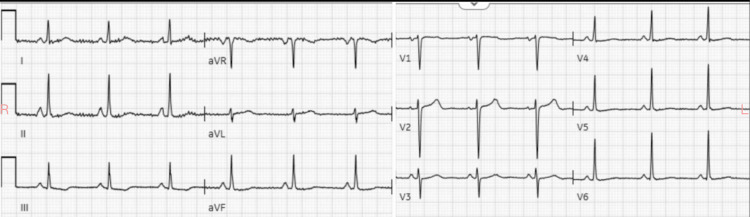
Patient's normal ECG days after presentation and similar to priors

The Poison Control Center was contacted and they recommended an assessment of labs to identify co-ingestion, which were all negative. They also suggested eight hours of observation and supportive care. The patient returned to a bradycardia of 40-50 beats per minute with improving mental status. She was holding conversations with staff and was completely alert and oriented. Psychiatry was consulted for attempted suicide. The patient remained bradycardic for approximately eight to nine hours and then returned to her baseline heart rate of approximately 75 beats per minute. The repeat ECG is shown in Figure 2.

The patient was medically cleared and admitted to the inpatient psychiatry unit for attempted suicide. The patient had a medical follow-up and was not found to be bradycardic again, with a heart rate ranging from 70 to 80 beats per minute.

## Discussion

In this case, a woman in her 50s presented with sinus bradycardia secondary to an overdose of alprazolam, clonazepam, and zolpidem. Oral benzodiazepines and zolpidem rarely have cardiovascular complications [2]. There have been some cases of benzodiazepine-induced bradycardia in the past; however, it is exceedingly rare [4,10,11]. Eizadi-Mood et al. in 2011 performed a retrospective analysis of 267 patients and showed that 5.25% developed bradycardia and only one patient required the treatment with atropine; they concluded that benzodiazepine toxicity does not have any significant risks of cardiovascular toxicity [4]. Arroyo et al. in 2012 described two cases where benzodiazepines, clonazepam, and alprazolam, respectively, induced first-degree atrioventricular (AV) block in two patients [10]. Mullins in 1999 described a case of a 28-year-old male who ingested 12 mg of alprazolam and developed a severe first-degree AV block with a PR of 527 and a heart rate of 58, which was reversed by flumazenil [12]. Maruyoshi et al. in 2017 described a case of clonazepam-induced sinus bradycardia with first-degree AV block in an elderly woman taking a therapeutic dosage [11]. In animal models, there has been prior research showing that benzodiazepines, specifically diazepam, can lower the inotropy of the heart; however, this did not affect the chronotropy [13]. There are peripheral low-affinity benzodiazepine receptors associated with calcium channels, primarily in neural tissue, but also in the heart [14]. Specific benzodiazepines, like diazepam, react with both the L-type and T-type calcium channels; while others, like clonazepam, preferentially bind only the T-type or transient calcium channels [14]. This would hypothetically cause increased AV blockade that could potentially be reversed by atropine, such as in our case. Certain benzodiazepines, specifically diazepam, and midazolam, have also been shown to block baroreflex control and sympathetic activity in the heart, which would also explain the patient’s bradycardia with relatively low blood pressure [15].

On the other hand, zolpidem has been associated with an increased incidence of acute myocardial infarction [9]. However, this was not the etiology of the bradycardia in our case, which presented with no chest pain and negative troponins. There have been a few case reports of zolpidem causing QT prolongation, but there was no reported bradycardia in these case reports [16]. In 2019, Sinha and Yadav performed a randomized control trial that showed that zolpidem at 10 mg doses lowered the heart rate of healthy males by approximately 5-8% while in non-rapid eye movement sleep, even though prior studies did not have similar findings [17,18]. They explain that zolpidem binds to the GABA-A receptor at the postsynaptic membrane of the rostral ventrolateral medulla, which inhibits neuronal outflow in the sympathetic system [17]. This explanation of bradycardia could also be reversed by atropine, which would block the parasympathetic input to the heart, as in our case.

Overall, whether it was the benzodiazepines, zolpidem, or both, our patient suffered from sinus bradycardia to approximately 35 beats per minute that responded to reversal with atropine. The decision to administer atropine initially was due to decreased mental status; however, when the patient once again became bradycardic, due to wearing off of the atropine, it was not administered again due to improving neurological status. Although flumazenil can be used to reverse both benzodiazepines, primarily, and zolpidem, the chronicity of the patient’s usage of both these medications was not known, and therefore it was not administered due to the high potential for serious adverse effects [19,20].

## Conclusions

Oral benzodiazepine and zolpidem overdoses can potentially present with bradycardia, which can be easily reversed with atropine. Although this patient was hemodynamically stable, due to the fact that the patient had impaired consciousness, atropine was chosen as an effective treatment for benzodiazepine- and/or zolpidem-induced bradycardia. It is important for the emergency physician to be aware of this potential side effect of benzodiazepine and/or zolpidem overdose, which have been previously thought to be more or less a benign overdose.
